# *DYRK1A* syndrome presenting with a familial exudative vitreoretinopathy (FEVR)-like retinovascular phenotype

**DOI:** 10.1080/13816810.2025.2503388

**Published:** 2025-05-22

**Authors:** Siying Lin, Eleanor Hay, Dorothy A. Thompson, Mariya Moosajee, Andrew R. Webster, Omar A. Mahroo, Robert H. Henderson, Gavin Arno

**Affiliations:** a Manchester Centre for Genomic Medicine, Saint Mary’s Hospital & Department of Ophthalmology, Manchester University NHS Foundation Trust, Manchester, UK; b Division of Evolution, Infection and Genomics, School of Biological Sciences, Faculty of Biology, Medicine and Health, University of Manchester, Manchester, UK; c National Institute of Health Research Biomedical Research Centre at Moorfields Eye Hospital, The UCL Institute of Ophthalmology, London, UK; d UCL Institute of Ophthalmology, University College London, London, UK; e Department of Clinical Genetics, Great Ormond Street Hospital for Children, London, UK; f Department of Clinical and Academic Ophthalmology, Sight and Sound Centre, Great Ormond Street Hospital for Children, London, UK; g UCL Great Ormond Street Institute of Child Health, University College London, London, UK; h Ocular Genomics and Therapeutics, The Francis Crick Institute, London, UK; i Department of Ophthalmology, St Thomas’ Hospital, London, UK; j Division of Research, Greenwood Genetic Center, Greenwood, South Carolina, USA

**Keywords:** DYRK1A, familial exudative vitreoretinopathy, intellectual disability, microcephaly

## Abstract

**Introduction:**

The DYRK1A gene plays a crucial role in central nervous system development, with haploinsufficiency leading to DYRK1A-related intellectual disability syndrome. Ocular manifestations are common in DYRK1A syndrome and include refractive error, strabismus and optic nerve hypoplasia. Retinal involvement, however is less frequently reported and remains uncharacterised.

**Methods:**

We conducted comprehensive ocular and systemic evaluations in two unrelated individuals with familial exudative vitreoretinopathy (FEVR)-like presentations and de novo DYRK1A variants. Genetic testing included whole genome sequencing with variant interpretation based on clinical guidelines.

**Results:**

Patient 1 had a previously reported recurrent pathogenic DYRK1A variant [c.1282C>T; p.(Arg428Ter)], whilst Patient 2 had a novel missense likely pathogenic variant [c.857T>C; p.(Leu286Pro)]. Both patients demonstrated systemic features consistent with DYRK1A syndrome.

**Discussion:**

These cases confirm vitreoretinal involvement as an associated finding in DYRK1A syndrome and highlight FEVR-like retinovascular abnormalities as a potential diagnostic clue for the condition in individuals with neurodevelopmental disorders.

## 1. Introduction

The *DYRK1A* gene encodes a member of the dual-specificity tyrosine phosphorylation-regulated kinase (DYRK) family, which is critical for central nervous system development, and plays a key role in neurogenesis, neural proliferation and differentiation, cell cycle regulation and synaptic plasticity (1). *DYRK1A* is located on chromosome 21q22.13, within the Down syndrome critical region, and exerts its effects in a dosage-sensitive manner. Trisomy driven overexpression of *DYRK1A* is implicated in the neuropathology of Down syndrome (2), whilst haploinsufficiency caused by heterozygous pathogenic variants results in *DYRK1A* syndrome, characterized by intellectual disability, autism spectrum disorder, distinctive facial features, and microcephaly (3). Ocular manifestations are observed in approximately two-thirds of affected individuals, primarily involving refractive error and strabismus. Retinal involvement is reported in around 5% of cases, with isolated reports of retinal detachment, retinal dystrophy, and peripheral retinal avascularity (4, 5).

This study reports two patients with a familial exudative vitreoretinopathy (FEVR)-like presentation associated with *DYRK1A* disease variants, consolidating this as a phenotypic feature of the condition.

## 2. Materials and methods

This study adhered to the Declaration of Helsinki and received ethical approval from Moorfields Eye Hospital and the Northwest London Research Ethics Committee (12/LO/0141). Written informed consent for participation and publication was obtained from the parents, who were also the legal guardians for both patients. Comprehensive clinical histories and in-depth phenotyping, including ophthalmic and systemic assessments, were conducted. Visual electrophysiology (Patient 1) followed a pediatric electroretinography protocol with periorbital skin electrodes at Great Ormond Street Hospital (GOSH-ERG) (6). Whole genome sequencing (WGS) was performed for both patients; with details provided in the supplemental information.

## 3. Results

### 3.1. Clinical findings

**Patient 1** is a 3-year-old female, the only child of unrelated Bengali parents. One of her two maternal half-brothers has autism, but there is no family history of ocular or developmental disorders. She was born at 37 weeks, weighing 1.8 kg (<0.4th centile, Z = −4.39), following induction of labor for intrauterine growth retardation. Prenatal imaging noted cerebellar abnormalities, and a day 5 postnatal MRI confirmed cerebellar vermian hypoplasia and additionally revealed right microphthalmia with intraocular hemorrhage and a left staphyloma, prompting a referral to ophthalmology.

**Figure 1.**
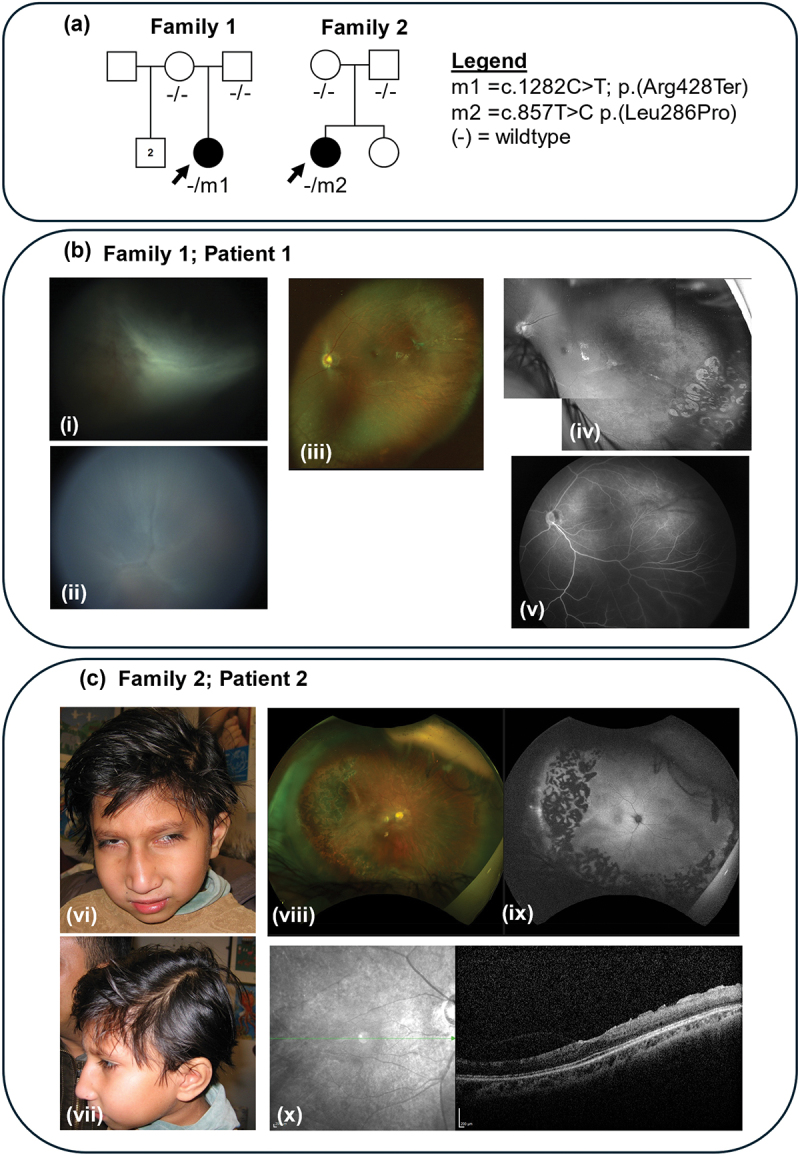
Family pedigrees and clinical findings for patients 1 and 2. (a) Family pedigrees for patients 1 and 2 (families 1 and 2 respectively) showing genotypes and familial segregation (b) retcam fundus photography of the right eye for patient 1 showing a fixed retinal Fold (i) and total retinal detachment (ii). Optos widefield pseudocolor imaging (iii) and green laser image (iv) of the left eye showing macular changes with epiretinal glial proliferation, straightened and temporally dragged vessels, and laser scars in the temporal retinal periphery. The brightness levels in the composite green laser image have been adjusted for optimal visual comparison. Retcam fluorescein angiography reveals areas of avascular retina and vascular leakage inferotemporally in the left eye (prior to laser photocoagulation)(v). (c) Clinical features of Patient 2 include deep-set eyes, upslanting palpebral fissures, long lashes, broad nasal tip, and a small chin, as shown in (vi) and (vii). Optos widefield pseudocolour (viii) and autofluorescence imaging (ix) of the right eye showing optic disc pallor, peripheral retinal fibrosis and scarring from panretinal photocoagulation treatment to areas of temporal retinal ischemia. An OCT scan of the right eye (x) shows retinal thinning.

On ophthalmic assessment, an objection to left eye occlusion was noted. The left eye was myopic, with a refraction of −7.50/-1.00 × 180° diopters; refraction was not possible on the right. Examination revealed right microphthalmia (axial length 13.6 mm at 1 month) with a fixed retinal fold and total retinal detachment; the left eye (axial length 17.8 mm at 1 month) showed macular dragging with an attached retina, features consistent with a FEVR-like retinovascular phenotype. Prophylactic retinal laser was performed to the left eye to reduce the risk of retinal detachment (Figure 1b). Electroretinography (ERG) with skin electrodes detected no response from the right eye, indicating severe generalized retinal dysfunction. A subnormal but reproducible skin ERG from the left eye was consistent with residual generalized retinal function. Flash visual evoked potentials (VEPs) demonstrated a robust and reproducible response from the left eye showing the left eye pathways were activating the striate cortex. The right eye VEP was small with atypical morphology, providing only equivocal evidence of visual pathway activation (Supplemental Results).

Systemic review revealed global developmental delay, with significant speech and language delay, and autistic tendencies. She had corrective surgery for pyloric stenosis at 6 weeks and had been treated for tonic-clonic epilepsy from 2 years. At the most recent examination, she remained of short stature (2nd centile Z = −1.79) with disproportionate microcephaly (<0.4th centile −4.74), right microphthalmia, prominent ears, café-au-lait macules over the abdomen and increased tone in arms and legs.

**Patient 2** (GC21304) is a 20-year-old female born to unrelated Bengali parents, with a healthy younger sister and no relevant family history. She initially presented to ophthalmology services at 6 years of age with a closed funnel retinal detachment in the left eye, which appeared tractional in origin. The right eye was myopic, with a refraction of −9.50/-2.25 × 010° diopters, and a left exotropia was noted. Examination and fluorescein angiography under anesthesia revealed 360° peripheral retinal vascular closure, a pale optic disc, and temporal retinal fibrosis in the right eye, consistent with a FEVR-like retinovascular phenotype. Prophylactic retinal laser was performed to the right eye to reduce the risk of retinal detachment. Both eyes subsequently developed cataracts; a cataract operation with intraocular lens insertion in the right eye improved visual acuity to 0.36 logMAR. A dense cataract remained in the left eye, which subsequently developed band keratopathy.

Neonatal issues included low birth weight (2.25 kg at 38 weeks’ gestation) and suspected Hirschsprung disease treated with a colostomy at 48 h of age, later reversed at 5 months. Her medical history included global developmental delay, microcephaly (<0.4th centile), and epilepsy. An MRI scan at age 7 showed mild cerebral atrophy. Dysmorphic facial features included deep-set eyes, upslanting palpebral fissures, long lashes, a broad nasal tip, and a small chin (Figure 1c).

## 4. Genetic findings

For Patient 1, genomic analysis identified only a single disease variant relevant to the patient’s phenotype: a *de novo* heterozygous variant in *DYRK1A* (NM_00139647721.2) c.1282C>T; p (Arg428Ter). This variant is absent from the gnomAD population database (v.4.1.0) and is predicted to result in premature protein truncation and loss of function. It has previously been associated with syndromic intellectual disability (7) and classified as pathogenic. No additional pathogenic variants were identified in genes associated with the patient’s systemic or ocular phenotype, including *KIF11* and other genes commonly implicated in microcephaly and chorioretinopathy syndromes, as well those linked to the FEVR spectrum.

For Patient 2, initial genomic analysis using the clinical pipeline did not identify any pathogenic variants in *KIF11* and other genes commonly implicated in microcephaly and chorioretinopathy syndromes, nor in genes associated with FEVR or inherited retinal disease. Further analysis focusing on rare variants [minor allele frequency of <0.001 in gnomAD and the Genomics England rare disease cohort], and predicted to be pathogenic by *in silico* tools, identified a single candidate variant: a *de novo* heterozygous variant in *DYRK1A* c.857T>C; p (Leu286Pro). This variant is absent from both gnomAD and the Genomics England rare disease cohort, with strong *in silico* pathogenicity predictions (Revel 0.9, AlphaMissense 1.0). Notably, a different amino acid substitution at the same codon, p (Leu286Phe) [previously annotated as p (Leu295Phe)], is a known pathogenic variant associated with syndromic intellectual disability (3). This variant was classified as likely pathogenic and is considered the molecular diagnosis responsible for the clinical presentation.

## 5. Discussion

*DYRK1A* syndrome is a well-established neurodevelopmental disorder caused by heterozygous variants in the *DYRK1A* gene leading to haploinsufficiency. It is characterized by a clinically recognizable phenotype, including moderate-to-severe developmental delay or intellectual disability, intrauterine growth retardation, microcephaly, autism spectrum disorder, characteristic facial features, delayed motor milestones, neonatal feeding difficulties, visual impairment, and seizures (8). *DYRK1A* syndrome is one of the most frequent monogenic neurodevelopmental disorders, accounting for approximately 0.1% to 0.5% of individuals diagnosed with intellectual disability and/or autism spectrum disorder (9).

Ocular findings are present in over 60% of patients with *DYRK1A* syndrome, with the most common being refractive errors (36%), enophthalmos (36%), strabismus (21%) and optic nerve abnormalities (20%) (5). Retinal involvement, including retinal detachment and retinal dystrophy, has been reported in a small number of cases, but remains poorly characterized (5). A recent case report described unilateral iris coloboma and anomalous peripheral retinal vasculature suggestive of FEVR in a patient with *DYRK1A* syndrome (4). This study, reporting a FEVR-like phenotype in two additional individuals, consolidates the FEVR spectrum as an associated feature of *DYRK1A* syndrome, expanding the phenotypic spectrum, and suggesting more extensive ocular involvement than previously recognized.

The retinal phenotype observed in our patients shows overlap with *KIF11*-related retinopathy and other forms of microcephaly and chorioretinopathy syndrome, which similarly present with microcephaly, microphthalmia, peripheral retinal avascularity, and tractional retinal detachments (10–12). However, *KIF11*-associated disease is also associated with chorioretinal atrophy—absent in our patients with *DYRK1A*-associated retinopathy—highlighting this as a potentially useful clinical marker to distinguish between the two.

FEVR is an inherited vitreoretinal disorder characterized by abnormal or incomplete vascularization of the peripheral retina, leading to a spectrum of clinical manifestations; milder cases may remain asymptomatic, whilst severe cases can result in significant vision loss due to complications including neovascularisation, retinal traction, folds, detachments, and vitreous hemorrhage (13). The genetic basis of FEVR has been linked to aberrant Wnt signaling, a pathway crucial for retinal development, maintenance and repair (14). *DYRK1A* has been shown to modulate the expression of canonical Wnt target genes, suggesting a potential mechanism linking *DYRK1A* dysfunction and FEVR (15).

Animal models have provided further insights into the role of *DYRK1A* in ocular development. *Dyrk1a*^±^ mice demonstrate microphthalmia, thinner retinas, reduced retinal ganglion cell numbers, abnormal retinal function on ERG, and defects in developmental retinal vascularization, with *Dyrk1a* proposed to play a key role in regulating endothelial cell proliferation and angiogenesis (16, 17). In zebrafish, *DYRK1A* orthologs are expressed in the central nervous system, retina, and endothelial cells during development, with *DYRK1A* kinase activity thought to be crucial for vascular formation via regulation of calcium signaling (18). These findings suggest that *DYRK1A* plays a critical role in ocular development and retinal vascularization, which may explain the retinal vascular anomalies and FEVR-like phenotype observed in patients with *DYRK1A* syndrome.

Recognising the FEVR spectrum as part of the *DYRK1A* phenotype emphasizes the importance of comprehensive ophthalmology evaluations in individuals with *DYRK1A* syndrome, potentially allowing earlier detection of retinal vascular anomalies and timely intervention to prevent associated complications. As patients may present with a FEVR-like phenotype as the initial clinical sign, *DYRK1A* should be included in FEVR gene panels to ensure accurate molecular diagnosis. Further research is needed to explore the molecular mechanisms underlying the diverse ocular and systemic manifestations of *DYRK1A* syndrome.

## Supplementary Material

Supplemental Material

## Data Availability

Research on the de-identified patient data used in this publication can be carried out in the Genomics England Research Environment subject to a collaborative agreement that adheres to patient led governance. All interested readers will be able to access the data in the same manner that the authors accessed the data. For more information about accessing the data, interested readers may contact research-network@genomicsengland.co.uk or access the relevant information on the Genomics England website: https://www.genomicsengland.co.uk/research.
