# Supplementary material for: DYRK1A syndrome presenting with a familial exudative vitreoretinopathy (FEVR)-like retinovascular phenotype
Source: Ophthalmic Genet. 2025 May 22;46(4):389–93. doi: 10.1080/13816810.2025.2503388 (PMC12312742; doi:10.1080/13816810.2025.2503388)
Supplement: Supplemental Material [file IOPG_A_2503388_SM6581.docx]

**Supplemental Material**

**Supplemental Methods – genetic testing**

Previous genetic testing for both patients included microarray-based comparative genomic hybridisation, which did not identify any clinically significant structural chromosomal abnormalities. Subsequently, trio whole genome sequencing was performed for both patients.

Patient 1 underwent whole genome sequencing (WGS) through the UK National Health Service (NHS) Genomic Medicine Service (GMS), whilst Patient 2 had WGS performed through the UK 100,000 Genomes Project (1). Bioinformatic analysis utilised a clinical pipeline, focusing on protein-altering variants within applied Genomics England PanelApp virtual gene panels consisting of disease genes associated with specific disease phenotypes (https://panelapp.genomicsengland.co.uk/) (2). For Patient 1, genomic analysis was performed using the following gene panels: Cerebral Malformations v8.2 (98 genes), Genetic Epilepsy Syndromes v3.0 (489 genes), Hereditary Ataxia and Cerebellar Anomalies - Childhood Onset (263 genes), Paediatric Disorders v24.2 (2295 genes), Severe Microcephaly v3.0 (165 genes), Retinal Disorders v3.0 (247 genes) and Structural Eye Disease v2.0 (127 genes). Patient 2 was analysed with the Posterior Segment Abnormalities gene panel v1.68 (202 genes).

**References**

1. The National Genomic Research Library v5.1. *Genomics England*. doi:10.6084/m9.figshare.4530893.v7

2. Martin AR, Williams E, Foulger RE, Leigh S, Daugherty LC, Niblock O, Leong IUS, Smith KR, Gerasimenko O, Haraldsdottir E, et al. PanelApp crowdsources expert knowledge to establish consensus diagnostic gene panels. *Nat Genet*. 2019;51(11):1560-5.

**Supplemental Results – Visual Electrophysiology (Patient 1)**


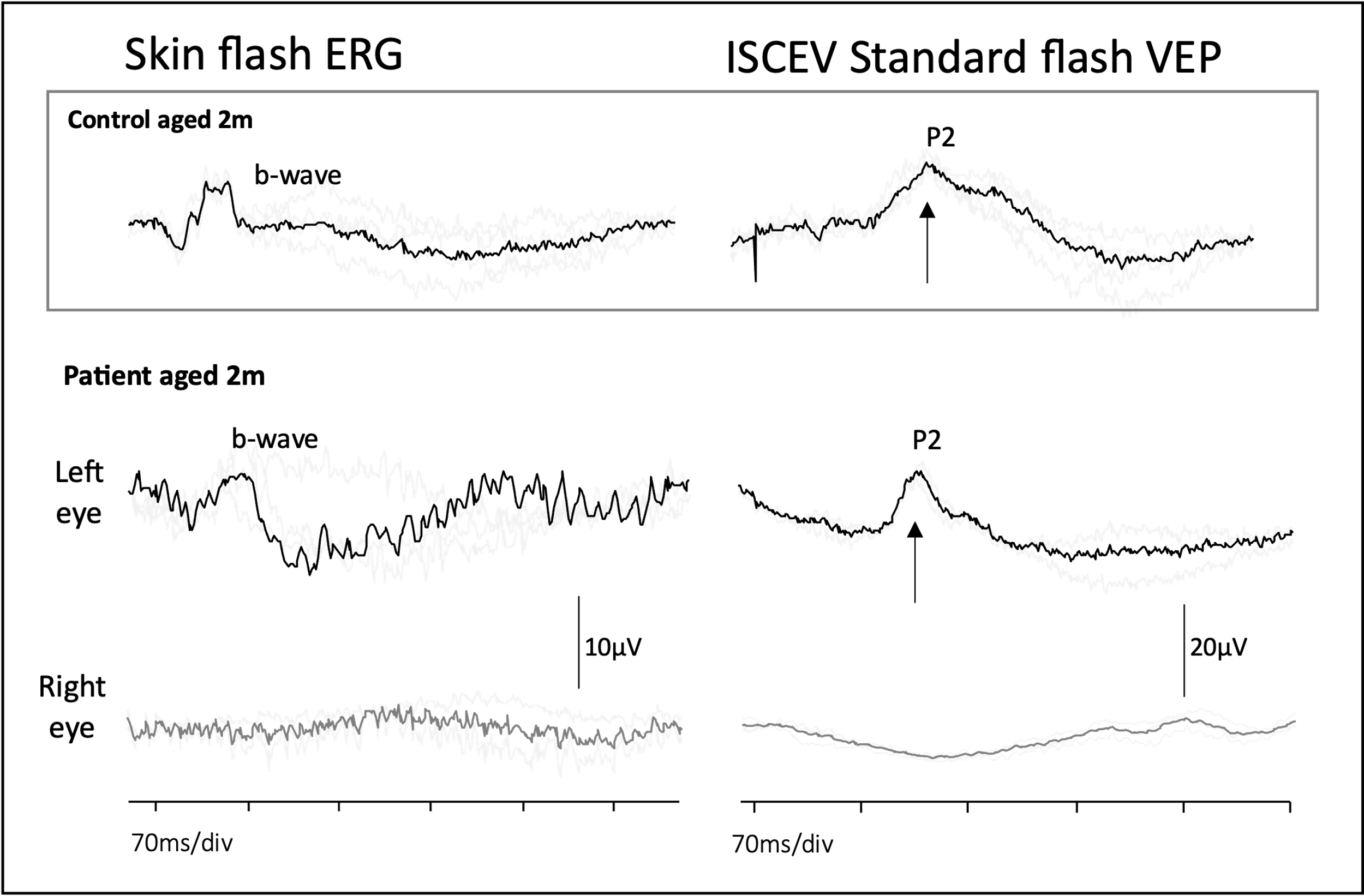


Electroretinography (ERG) and visual evoked potentials (VEPs) in Patient 1 at age 2 months indicate severe retinal dysfunction in the right eye, but evidence of left eye retinal function and intact left eye visual pathway activation. Examples from a healthy control, aged 2 months, shown in the top box.
